# Prevalence of Musculoskeletal Disorders and Associated Risk Factors Among Leather Factory Workers in Chengalpattu District, Tamil Nadu, India: A Cross-Sectional Study

**DOI:** 10.7759/cureus.95931

**Published:** 2025-11-02

**Authors:** Gowtham S, Ananth Eshwar, Ilamilaval Thozhanenjan, Priyanka Vittal

**Affiliations:** 1 Community Medicine, Sree Balaji Medical College And Hospital, Chennai, IND; 2 Community Medicine, Sree Balaji Medical College and Hospital, Chennai, IND; 3 Community Medicine, Indira Gandhi Government Medical College, Nagpur, IND

**Keywords:** ergonomics factors, leather industry, musculoskeletal disease, occupational hazards, occupational health

## Abstract

Background

The leather industry is a significant economic sector in Tamil Nadu, contributing over 40% of India's leather exports. However, leather factory workers face substantial occupational health risks due to chemical exposures, repetitive tasks, and poor ergonomic conditions.

Objective

This study aimed to determine the prevalence of musculoskeletal disorders (MSDs) and identify associated risk factors among leather factory workers in the Chengalpattu district, Tamil Nadu.

Methods

A cross-sectional study was conducted from July to December 2024 among 300 workers from 10 randomly selected leather factories. Data were collected using a pre-tested semi-structured questionnaire and the Modified Nordic Questionnaire. Statistical analysis included chi-square tests, odds ratios, and logistic regression using SPSS v25 (IBM Corp., Armonk, USA).

Results

The prevalence of musculoskeletal disorders was 243 (81%). Significant risk factors identified through logistic regression included age >30 years (adjusted odds ratio (AOR)=1.62, 95% CI: 1.24-3.85, p=0.03) and illiteracy (AOR=2.98, 95% CI: 1.43-6.20, p=0.003). Alarming workplace conditions were observed, with 213 (71%) workers not using personal protective equipment, 230 (76.7%) exposed to leather dust, and 191 (63.7%) working in poorly ventilated environments.

Conclusion

The high prevalence of musculoskeletal disorders necessitates immediate occupational health interventions, including ergonomic modifications, mandatory use of personal protective equipment (PPE), improved ventilation, worker education programs, and regular health surveillance targeting high-risk workers.

## Introduction

One of the oldest and most famous industries of India, the leather industry, contributes heavily to the industrial growth, export earnings, and employment generation of the country. With some of the biggest tanneries and production units, having major hubs at Chennai, Ambur, Ranipet, Vaniyambadi, Trichy, and Dindugal, Tamil Nadu dominates India's leather industry with more than 40% of the country's exports [1]. Leather factories are significant to the world economy, and the business of leather goods was around USD 407.92 billion in 2023 and is estimated to be USD 629.65 billion in 2030 [2].

Globally, the leather sector employs directly around 500,000 individuals in the formal factory setting, with an additional million in informal and cottage industry operations [3]. China, India, Brazil, Italy, and Bangladesh are the world's leading countries, accounting for over 65% of the global leather production [4]. India accounts for approximately 13% of the total world leather production. India is also the second-largest producer and consumer of leather footwear. Some of the highest production states in India include Tamil Nadu, West Bengal, Uttar Pradesh, Maharashtra, Punjab, Karnataka, Madhya Pradesh, Haryana, Kerala, Rajasthan, and Jammu & Kashmir [5]. Tamil Nadu's leather industry, supported by its large livestock base [6]. 

Morbidities, including dermatitis and respiratory diseases, are caused by chemical exposure, with mortality enhanced by occupational cancers and cardiovascular disease [3].

International studies have indicated high prevalence rates of musculoskeletal diseases (40-70%) in leather workers compared to the general population [7].

Musculoskeletal disorders are one of the major occupational morbidities, primarily caused by ergonomic factors like repetitive motion, awkward postures, heavy load lifting, and prolonged standing. Low back pain (45-65%), upper extremity disorders (30-50%), and neck/shoulder pathologies (25-40%) were observed among factory workers engaged in the manufacture of leather articles [8].

The most important risk factors are: repetitive movements - particularly in cutting, sewing, and finishing operations [9]; awkward postures - most of the time, employees will be in non-ergonomic bending or standing positions [10]; manual handling - excessive handling of goods and hides during lifting and carrying [11]; and poor design of workstations - non-ergonomic work surfaces and tools [12].

The incidence of lower back pain was documented in a cross-country study in large leather-producing nations (Italy, Brazil, China, India, Mexico) as 47-68%, neck/shoulder pain 42-58%, and wrist/hand pain 38-52% [13]. 

Although this sector holds significant economic value, there is insufficient research examining occupational health hazards faced by Indian leather industry workers, especially those in the Chengalpattu district. Therefore, this study aimed to estimate the prevalence of musculoskeletal disorders and their risk factors among leather factory workers.

## Materials and methods

Study area and population

The study was carried out in leather factories in the Chengalpattu district from July to December 2024. The study participants include workers employed for more than one year in a leather factory. Administrative staff working at the leather factory were excluded.

Sample size and sampling method

A study done by Judyangel in Ambur, Tamil Nadu, reported an MSD prevalence of 37% [14]. Considering this as the prevalence (p) and applying the formula \begin{equation} n = \frac{Z^2pq}{d^2} \end{equation} and Z=1.96 for 95% CI, p=37%, q=(100-p)=63% and allowable error (d)=5%, the sample size was 249. Allowing a 15% non-response rate, the required sample size was rounded off to 300. The leather factory list was taken from the Chengalpattu district tannery association. To attain the sample size, 10 factories were randomly selected from the list by the lottery sampling method. In each factory, there are approximately 50-60 workers. Thirty workers from each factory were selected by simple random sampling. On getting permission from the factory head, all workers who had given consent and were under the inclusion criteria were taken as study participants. If the matching random participant was not available or was not willing, the next participant was taken into account.

Data collection

After a small introduction, the chief investigator provided an explanation of the study's purpose to the participants. Informed consent was received from the study respondents in Tamil and English. The participants were informed that all information gathered would be kept confidential. The data was gathered from the subject through a pre-tested, semi-structured questionnaire (see Appendices) and was recorded through face-to-face interviews. Data was collected from the participants in Tamil is translated into English. The Modified Nordic Questionnaire was used to estimate the prevalence of musculoskeletal disorders [15]. Two sections made up the questionnaire: the first section of the questionnaire was for ascertaining the prevalence of musculoskeletal disorders; in case of a negative response in the first section, the subject did not have to proceed to the other sections. The second section determined work interference, severity, and duration. The B.G. Prasad scale was used to assess the socioeconomic status of the participants [16].

Data analysis

Data was entered in Microsoft Excel (Microsoft Corporation, Redmond, USA) and analysed using IBM SPSS v25 (IBM Corp., Armonk, USA). Descriptive data are presented in frequencies and percentages. Chi-square tests and odds ratios were used to examine the association of musculoskeletal disorders. Variables that were statistically significant in the bivariate analysis were included in the logistic regression analysis.

## Results

Table 1 shows the sociodemographic details of the study participants. Among 300 participants, 208 (69.3%) were ≤30 years of age, 199 (66.3%) were male, 121 (40.3%) were Illiterate, 194 (64.7%) had work experience ≤ 10 years, 213 (71%) did not use personal protective equipment (PPE), 230 (76.7%) were exposed to leather dust, and 191 (63.7%) participants work in poor ventilated work environment.

**Table 1:** Sociodemographic Details of Study Participants Data are presented as n (%), where n represents the number of responses in each variable and (%) indicates the percentage of participants, respectively. Socioeconomic status was classified according to the B.G. Prasad Scale as follows: Class I – Upper Class (₹8592 and above); Class II – Upper Middle Class (₹4269–₹8591); Class III – Middle Class (₹2578–₹4295); Class IV – Lower Middle Class (₹1289–₹2577); and Class V – Lower Class (below ₹1289) [16]. PPE: personal protective equipment

S. No	Variable	Frequency	Percentage
		(n=300)	(%)
1.	AGE		
	>30 years	92	30.7
	≤30 years	208	69.3
2.	GENDER		
	Female	101	33.7
	Male	199	66.3
3.	RELIGION		
	Hindu	98	32.7
	Muslim/Christian	202	67.3
4.	EDUCATION		
	Illiterate	121	40.3
	Literate	179	59.7
5.	BMI		
	Obesity/Overweight	128	42.7
	Underweight	33	11
	Normal	139	46.3
6.	MARITAL STATUS		
	Married	84	28
	Single	216	72
7.	SOCIOECONOMIC STATUS		
	Class III/Class IV	78	26
	Class I/Class II	222	74
8.	WORK EXPERIENCE		
	>10 years	106	35.3
	≤10 years	194	64.7
9.	SMOKING		
	Yes	133	44.3
	No	167	55.7
10.	DRINKING		
	Yes	142	47.3
	No	158	52.7
11.	TOBACCO CHEWING		
	Yes	65	21.7
	No	235	78.3
12.	COMORBIDITIES		
	Yes	143	47.7
	No	157	52.3
13.	PPE USE		
	No	213	71
	Yes	87	29
14.	EXPOSURE TO LEATHER DUST		
	Yes	230	76.7
	No	70	23.3
15.	VENTILATION		
	Poor	191	63.7
	Good	109	36.3
16.	CHEMICAL EXPOSURE		
	Yes	82	27.3
	No	218	72.7

Table 2 shows the association between sociodemographic characteristics and musculoskeletal disorders. On bivariate analysis, participants aged >30 years were significantly more likely to experience musculoskeletal disorders compared to ≤30 years (OR 3.24, 95% CI 1.79-5.87, p<0.05). Among the participants, Illiterate workers (90.9%) experienced musculoskeletal disorders, and this was found to be statistically significant with p<0.05 and an odds ratio of 3.45 (95% CI 1.71-6.99). Other factors that were statistically significant with p<0.05 were being married, with an odds ratio of 3.54 (95% CI 1.94-6.45); work experience >10 years, with an odds ratio of 2.36 (95% CI 1.19-4.70); and presence of comorbidities, with an odds ratio of 1.89 (95% CI 1.04-3.45).

**Table 2:** Association Between Sociodemographic Characteristics and Musculoskeletal Disorder Data are presented as n (%), where n represents responses and (%) indicates the percentage of participants. The chi-squared test and odds ratio were used to test the association at a 95% CI. *p<0.05: statistically significant. 95% CI - 95% confidence interval. Socioeconomic status was classified according to the B.G. Prasad Scale as follows: Class I – Upper Class (₹8592 and above); Class II – Upper Middle Class (₹4269–₹8591); Class III – Middle Class (₹2578–₹4295); Class IV – Lower Middle Class (₹1289–₹2577); and Class V – Lower Class (below ₹1289) [16].

Sociodemographic and Other Variables	Musculoskeletal Disorder		Odds Ratio	95% CI	Chi-Square test	P value*
	Present Absent					
AGE						
>30 years	181 (87)	27 (13)	3.24	1.79-5.87	15.96	0.000*
≤30 years	62 (67.4)	30 (32.6)	Reference	-	-	-
GENDER						
Female	85 (84.2)	16 (15.8)	1.37	0.73-2.60	0.98	0.32
Male	158 (79.4)	41 (20.6)	Reference	-	-	-
RELIGION						
Hindu	80 (81.6)	18 (18.4)	1.06	0.57-1.97	0.03	0.84
Muslim/Christian	163 (80.7)	39 (19.3)	Reference	-	-	-
EDUCATION						
Illiterate	110 (90.9)	11 (9.1)	3.45	1.71-6.99	12.93	0.001*
Literate	133 (74.3)	46 (25.7)	Reference	-	-	-
BMI						
Obese/Overweight	102 (79.7)	26 (20.3)	0.86	0.46-1.58	0.23	0.62
Underweight	27 (81.8)	6 (18.2)	0.98	0.36-2.64	0.00	0.97
Normal	114 (82)	25 (18)	Reference	-	-	-
MARITAL STATUS						
Married	188 (87)	28 (13)	3.54	1.94-6.45	18.26	0.000*
Single	55 (65.5)	29 (34.5)	Reference	-	-	-
SOCIOECONOMIC STATUS						
Class III/Class IV	62 (79.5)	16 (20.5)	0.87	0.46-1.67	0.15	0.69
Class I/Class II	181 (81.5)	41 (18.5)	Reference	-	-	-
WORK EXPERIENCE						
>10 years	94 (88.7)	12 (11.3)	2.366	1.19-4.70	6.28	0.012*
≤10 years	149 (76.8)	45 (23.2)	Reference	-	-	-
SMOKING						
Yes	110 (82.7)	23 (17.3)	1.22	0.68-2.19	0.45	0.5
No	133 (79.6)	34 (20.4)	Reference	-	-	-
DRINKING						
Yes	114 (80.3)	28 (19.7)	0.91	0.514-1.63	0.09	0.76
No	129 (81.6)	29 (18.4)	Reference	-	-	-
TOBACCO CHEWING						
Yes	58 (89.2)	7 (10.8)	2.23	0.96-5.20	3.65	0.56
No	185 (78.7)	50 (21.3)	Reference	-	-	-
COMORBIDITIES						
Yes	123 (86)	20 (14)	1.89	1.04-3.45	4.46	0.03*
No	120 (76.4)	37 (23.6)	Reference	-	-	-
PPE USE						
No	178 (83.6)	35 (16.4)	1.72	0.94-3.15	3.14	0.07
Yes	65 (74.7)	22 (25.3)	Reference	-	-	-
EXPOSURE TO LEATHER DUST						
Yes	190 (82.6)	40 (17.4)	1.52	0.80-2.90	1.65	0.19
No	53 (75.7)	17 (24.3)	Reference	-	-	-
VENTILATION						
Poor	157 (82.2)	34 (17.8)	1.23	0.68-2.23	0.49	0.48
Good	86 (78.9)	23 (21.1)	Reference	-	-	-
CHEMICAL EXPOSURE						
Yes	190 (80.2)	47 (19.8)	0.76	0.36-1.61	0.50	0.47
No	53 (84.1)	10 (15.9)	Reference	-	-	-

Table 3 describes the logistic regression analysis to find the predictors of musculoskeletal disorders. It was observed that those aged >30 years (adjusted odds ratio (AOR) 1.62, 95% CI 1.24-3.85) were found to have a statistically significant association with musculoskeletal disorders. 

**Table 3:** Logistic Regression Analysis of Musculoskeletal Disorder and Related Variables Data are presented as n (%), where n represents responses and (%) indicates the percentage of participants. Logistic regression was used to test the association at a 95% CI. **p<0.05: statistically significant 95% CI: 95% confidence interval; AOR: adjusted odds ratio

Sociodemographic and Other Variables	Adjusted Odds Ratio	95% CI	P value**
AGE			
>30 years	1.62	1.24-3.85	0.03**
≤30 years	Reference	-	-
EDUCATION			
Illiterate	2.98	1.43-6.20	0.07
Literate	Reference	-	-
MARITAL STATUS			
Married	2.31	1.05-5.08	0.24
Single	Reference	-	-
COMORBIDITIES			
Yes	0.88	0.43-1.81	0.74
No	Reference	-	-
WORK EXPERIENCE			
>10 years	1.46	0.66-3.23	0.35
≤10 years	Reference	-	-

## Discussion

Prevalence of musculoskeletal disorders

The primary objective of the study was to determine the prevalence of musculoskeletal disorders. In that aspect, the prevalence of musculoskeletal disorders in this study is 243 (81%). Similarly, in a 2020 study by Kanniappan and Palani in Chennai found a prevalence of 88%, indicating a high prevalence of musculoskeletal disorders [17]. In a 2024 study by Rmadi et al. in South Tunisia, the prevalence of musculoskeletal symptoms was reported as 65.5% for men and 72.4% for women, indicating a significant occurrence of musculoskeletal disorders among these workers [18]. A study by Wang et al. in China reported an overall prevalence rate of 79.7% for work-related musculoskeletal disorders across four manufacturing factories in China [19]. A study by Silva and Teixeira in Minas Gerais found a higher prevalence of musculoskeletal complaints among leather factory workers - 40.0% in males and 24.1% in females [20]. A study by Lim et al. in Malaysia found a 45.4% prevalence rate of musculoskeletal disorders among factory workers [21]. A 2022 study by Jin et al. in China reported a 41.0% prevalence of lower extremity musculoskeletal disorders across four manufacturing factories in China [22].

The high prevalence rate in various countries is indicative of similar occupational risks in manufacturing and leather factories. The workforce involves risk exposures such as repetitive movements, prolonged standing or sitting, poor postures, and manual handling activities that, by nature, put them at risk of musculoskeletal disorders. Differences between the studies are due to differences in study methodologies, such as study tools, inclusion criteria, and demographic differences.

Risk factors associated with MSDs

In the present study, the risk factor found to be associated with musculoskeletal disorders among leather factory workers was age. A 2021 study by Leite et al. found age was associated with musculoskeletal disorders among footwear industry workers [23]. A study done by Karthikeyan et al. among workers aged 25 to 60 years highlighted that musculoskeletal disorders risk is prevalent across this age range in the leather garments manufacturing industry, emphasising the need for ergonomic awareness and interventions [24]. A 2019 study by Putri noted that the majority of workers were under 25 years old, indicating a potential demographic factor in musculoskeletal complaints [25]. A 2022 study by Jin in China found that being more than 41 years of age was associated with an increased risk of musculoskeletal disorders among workers [22]. A study by Rahman et al highlighted that older leather factory workers faced increased risks of musculoskeletal disorders due to declining physical capacities, such as strength and endurance, while the physical demands of manual handling tasks remained unchanged, exacerbating health concerns [26].

The differences in age-related MSD risk across studies may be influenced by the nature of work, worker demographics, and ergonomic conditions. Older workers are generally at a higher risk due to physiological decline, but young workers can also be affected if exposed to poor working conditions without adequate training or support.

Recommendations

We recommend implementing workplace modifications, such as adjustable workstations and providing lifting devices**, **to prevent repetitive motion and prolonged standing. Regular ergonomic inspection must be done to identify and correct work postures. Mandate the use of PPE. Improve workplace ventilation systems to reduce exposure to leather dust and chemical hazards. Offer mandatory training programs in proper work methods, proper stance, and safe handling practices. Establish regular medical screening programs with a focus on employees with well-identified risk factors (age >30 years, work experience >10 years, presence of comorbidities). Institute job rotation, mandatory rest breaks, and reduce long-standing/sitting periods in order to avoid repetitive strain injuries. Supplement studies to assess the psychological impacts of occupational stressors and devise comprehensive occupational health policies appropriate for Tamil Nadu's leather industry.

Limitations

This cross-sectional study design limits the ability to establish causal relationships between risk factors and musculoskeletal disorders. The study was confined to Chengalpattu district, which may limit its generalizability to leather workers in other regions with different working conditions. Self-reported data through questionnaires may be subject to recall bias and social desirability bias.

## Conclusions

A high prevalence of 81% of musculoskeletal disorders was noted in this cross-sectional study among 300 leather factory workers in the Chengalpattu district, Tamil Nadu. The research showed alarming working conditions, where 71% of the employees were not in PPE, 76.7% were subjected to leather dust, and 63.7% were subjected to poor ventilation. In spite of high incidences of musculoskeletal disorders comparable to international research in manufacturing industries, working conditions like ergonomics deficiency, unsatisfactory working conditions, repetitive movements, and long standing are causative factors for this occupational health epidemic. These results demonstrate the necessity for widespread occupational health interventions in the leather industry, such as mandatory ergonomic evaluation, ongoing health monitoring programs, modifications in workplaces, and education programs for workers. Further longitudinal studies are suggested to estimate the long-term effect of interventions and to form evidence-based recommendations tailored to the leather production setting.

## Appendices

Questionnaire

Sociodemographic Information

1. Gender: • Male • Female

2. Age (in complete years): __________ years

3. Marital Status: • Single • Married • Divorced • Widow/Widower

4. Religion: • Hindu • Christian • Muslim

5. Monthly family income: __________

6. Number of family members: __________

7. Per capita income: __________

8. Socioeconomic Status (BG Prasad Scale): • Upper Class • •9414 and above • Upper Middle Class • •4707•9413 • Middle Class • •2824•4706 • Lower Middle Class • •1412•2823 • Lower Class • Below •1412

9. Educational Qualification: • Illiterate • Primary Class • High School • Higher Secondary School • Graduate • Postgraduate

10. Height (in cm): __________

11. Weight (in kg): __________

12. BMI: __________

Personal Details

1. Smoking: • Yes • No

2. Drinking: • Yes • No

3. Tobacco chewing: • Yes • No

4. Comorbidities: • Diabetes • Hypertension • Others: __________

5. Skin problems: • Yes • No

Professional Details

1. Work experience (in years): __________

2. PPE use: • Yes • No

3. Exposure to leather dust: • Yes • No

4. Ventilation at workplace: • Poor • Good

5. Chemical exposure: • Yes • No

6. Periodic examination: • Yes • No
